# Investigation of protein synthesis in *Drosophila* larvae using puromycin labelling

**DOI:** 10.1242/bio.026294

**Published:** 2017-06-22

**Authors:** Lisa P. Deliu, Abhishek Ghosh, Savraj S. Grewal

**Affiliations:** Clark H Smith Brain Tumour Centre, Arnie Charbonneau Cancer Institute, Alberta Children's Hospital Research Institute, and Department of Biochemistry and Molecular Biology Calgary, University of Calgary, Calgary, Alberta T2N 4N1, Canada

**Keywords:** *Drosophila*, mRNA translation, Protein synthesis, Nutrients, TOR kinase, dMyc, Hypoxia, Heat shock

## Abstract

Translational control of gene expression is an important regulator of growth, homeostasis and aging in *Drosophila*. The ability to measure changes in protein synthesis in response to genetic and environmental cues is therefore important in studying these processes. Here we describe a simple and cost-effective approach to assay protein synthesis in *Drosophila* larval cells and tissues. The method is based on the incorporation of puromycin into nascent peptide chains. Using an *ex vivo* approach, we label newly synthesized peptides in larvae with puromycin and then measure levels of new protein synthesis using an anti-puromycin antibody. We show that this method can detect changes in protein synthesis in specific cells and tissues in the larvae, either by immunostaining or western blotting. We find that the assay reliably detects changes in protein synthesis induced by two known stimulators of mRNA translation – the nutrient/TORC1 kinase pathway and the transcription factor dMyc. We also use the assay to describe how protein synthesis changes through larval development and in response to two environmental stressors – hypoxia and heat shock. We propose that this puromycin-labelling assay is a simple but robust method to detect protein synthesis changes at the levels of cells, tissues or whole body in *Drosophila*.

## Abstract (precis)

**Summary:** This paper describes a simple approach, using puromycin-labelling of nascent peptides, to assay protein synthesis in response to growth and stress signals in *Drosophila* larvae.

## INTRODUCTION

*Drosophila* is an excellent genetic model system for studying animal physiology, growth and development (Grewal, 2009; Partridge et al., 2011; Andersen et al., 2013; Padmanabha and Baker, 2014; Parsons and Foley, 2016). Over the last few decades, the versatility of *Drosophila* genetics has led to the identification of signalling pathways and gene expression networks important for normal growth, development and aging. Moreover, the amenability of *Drosophila* to biochemical analyses has allowed an understanding of how these networks regulate cellular biochemistry and physiology.

Many genes and signalling pathways that regulate protein synthesis have been shown to contribute to growth, stress responses, immune responses and aging. Developing methods to measure protein synthesis in *Drosophila* is therefore important in studying these regulators. Two classic methods to measure translation are polysome profiling and radioactive amino acid labelling of newly synthesized proteins. However, both have their drawbacks for analyzing protein synthesis in *Drosophila* – polysome profiling requires large amounts of material making it difficult to analyze specific larval cells or tissues, while radioactive amino acid labelling requires additional laboratory protocols and procedures to deal with radioactive samples. Moreover, neither approach can be used to analyze protein synthesis *in situ* in specific cells or tissues.

Here we present a simple, low cost assay to measure protein synthesis in *Drosophila* larval cells and tissues. This assay is based on a previously described puromycin labelling assay (the SUnSET assay) (Schmidt et al., 2009). Puromycin is an aminoacyl-tRNA analog that, when added to cells at low concentrations, can be incorporated into nascent peptides which then leads to termination of translation of these peptides (Nathans, 1964; Nakano and Hara, 1979; Hansen et al., 1994). By using an anti-puromycin antibody, these newly synthesized puromycin-labelled peptides can be detected by standard immunochemical methods, and the amount of puromycin labelling hence provides a measure of nascent protein synthesis. This approach has been increasingly used to monitor protein synthesis in mammalian cells (e.g. Goodman et al., 2011; Cook et al., 2014; Dalet et al., 2017). Here we show it can be applied to measure mRNA translational changes in larval tissues in response to environmental and genetic manipulations.

## RESULTS AND DISCUSSION

### Measuring protein synthesis during larval development

**Fig. 1.**
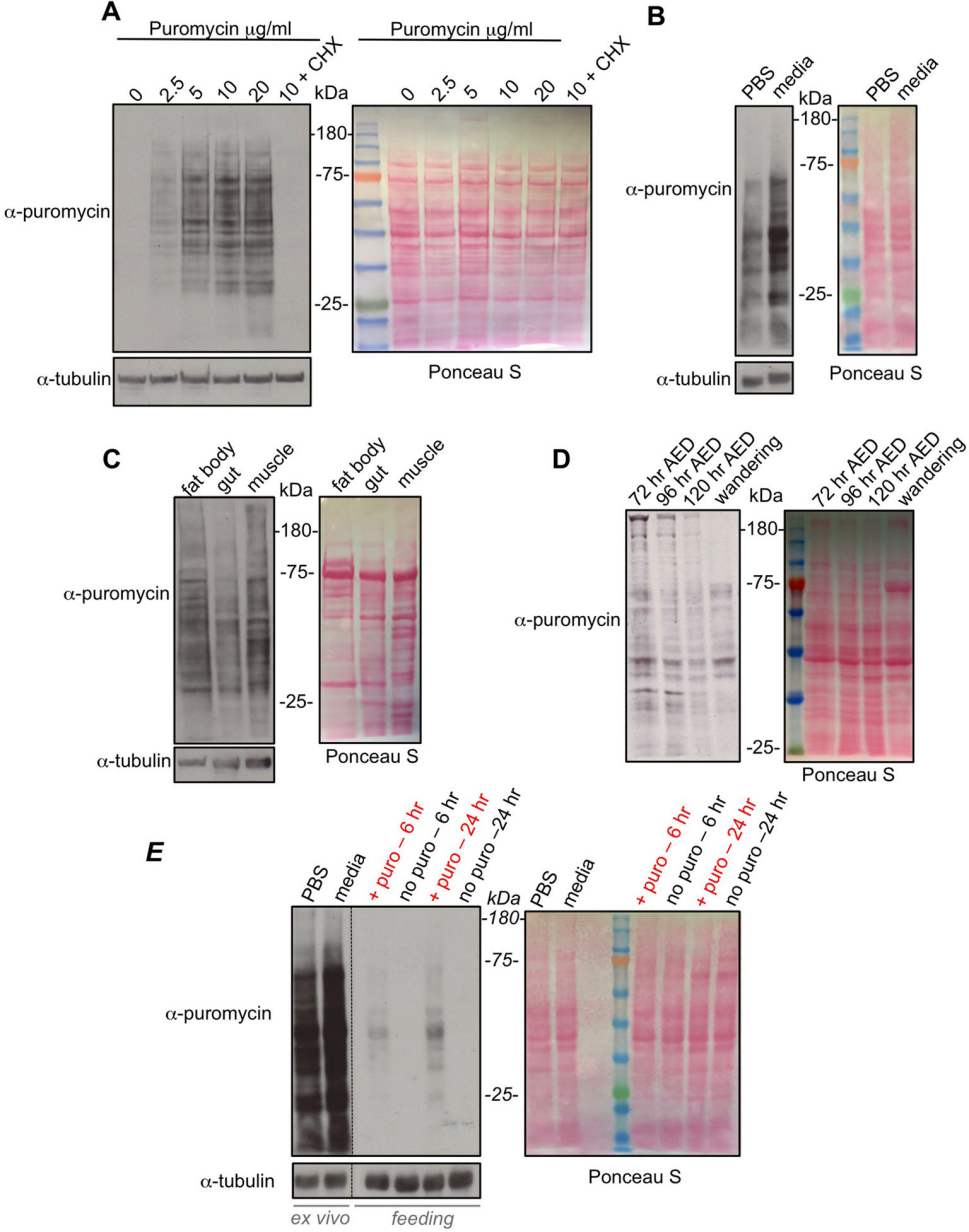
**Puromycin labelling to measure protein synthesis during larval development.** (A) Whole inverted third instar larvae were incubated in increasing amounts of puromycin (5 µg/ml), or with puromycin (5 µg/ml)+cycloheximide (CHX, last lane) together, for 40 min. Equal amounts of whole larval protein extracts were then analyzed by western blotting. Left, western blot with either anti-puromycin, or anti-tubulin antibodies. Right, Ponceau S staining showing total protein levels. (B) Whole inverted larvae were incubated in either PBS+puromycin (5 µg/ml) or Schneider's media+puromycin (5 µg/ml) for 40 min. Equal amounts of whole larval protein extracts were then analyzed by western blotting. Left, western blot with either anti-puromycin, or anti-tubulin antibodies. Right, Ponceau S staining showing total protein levels. (C) Whole inverted third instar larvae were incubated in Schneider's media+puromycin (5 µg/ml) for 40 mins. Larval tissues were then isolated and analyzed by western blotting. Left, western blot with either anti-puromycin, or anti-tubulin antibodies. Right, Ponceau S staining showing total protein levels. (D) Larvae at different stages in development (72 h AED, 96 h AED, 120 h AED and wandering stage) were inverted and incubated in Schneider's media+puromycin (5 µg/ml) for 40 min. Equal amounts of whole larval protein extracts were then analyzed by western blotting. Left, western blot with anti-puromycin. Right, Ponceau S staining showing total protein levels. (E) Comparing *ex vivo* versus *in vivo* feeding for puromycin labelling. For the *ex vivo* experiments, third instar larvae were inverted and incubated in either PBS+puromycin (5 µg/ml) or Schneider's media+puromycin (5 µg/ml) for 40 min. For the feeding experiments, third instar larvae were transferred to either normal food (no puro) or normal food supplemented with 25 µg/ml of puromycin (+ puro) for either 6 or 24 h. For both the *ex vivo* and *in vivo* samples, equal amounts of whole larval protein extracts were then analyzed by western blotting. Left, western blot with either anti-puromycin, or anti-tubulin antibodies. Right, Ponceau S staining showing total protein levels. Note, the vertical dotted line in the western blots indicates where the blot was spliced to remove an empty lane and the molecular weight ladder lane (see Ponceau S staining). All experiments were carried out using *w^1118^* larvae.

We began by establishing conditions in which we could obtain reliable labelling of nascent peptides by puromycin. We first tried an *ex vivo* labelling approach. Whole larvae can be inverted and their tissues can be maintained alive and metabolically active in media or buffer for several hours. This approach is widely used to perform BrdU or dye labelling of larval tissues in order to measure processes such as cell cycle progression, autophagy and lipid storage. We used this approach to measure protein synthesis. We inverted and then incubated whole third instar larvae in Schneider's media containing increasing amounts of puromycin for 40 min. We found that incorporation of puromycin into peptides/proteins increased progressively with higher concentrations of puromycin (Fig. 1A). Importantly, these effects were abolished if we also co-incubated tissues with cycloheximide, indicating that the puromycin incorporation was indeed a measure of protein synthesis. We also carried out experiments in which we performed the puromycin labelling in both the presence and absence of bortezomib, a proteasome inhibitor. We found that bortezomib had no effect on puromycin labelling (Fig. S1). From this, we infer that although puromycin incorporation leads to termination of the translation of labelled peptides, any potential proteasomal degradation of these labelled peptides does not confound the assay.

We also compared the effects of carrying out the puromycin labelling in media versus phosphate-buffered saline (PBS). We found that incorporation of puromycin did occur when larval tissues were incubated with PBS, although at a lower level than with incubation in Schneider's media (Fig. 1B). This may be because the lack of amino acids may either limit translation or may lead to loss of nutrient-dependent signalling pathways such as the TORC1 kinase pathway. It is worth noting that the levels of amino acids and glucose in Schneider's media are approximately the same as the levels measured in larval hemolymph (Cheng et al., 2011; Pasco and Léopold, 2012). Hence, although this is an *ex vivo* assay, by using Schneider's media for the labelling period, we are approximating some of the nutrient conditions *in vivo*.

In our assays, we relied on lysis of whole larvae for our western blots. We therefore next compared puromycin incorporation in different larval tissues. We carried out the puromycin labelling as normal and then isolated specific tissues and carried out western blots. We found robust puromycin incorporation in the three tissues we tested – fat body, gut and muscle – suggesting that the assay conditions probably allow for measurement of protein synthesis in all larval tissues (Fig. 1C).

We next compared protein synthesis levels at different stages of larval development. We found that protein synthesis levels were highest in larvae examined 72 h after egg deposition (AED) and then gradually declined throughout the remainder of larval development until wandering stage (Fig. 1D).

Together these data indicate that short-term *ex vivo* labelling of newly synthesized peptides provides an effective way to measure translation in larval tissues and whole animals during larval development. Another potential approach is to use *in vivo* labelling of nascent peptides with puromycin to measure new protein synthesis. To try this, we fed larvae food mixed with puromycin and then compared the labelling of peptides by this method with the *ex vivo* approach described above. We initially found that feeding larvae 5 µg/ml of puromycin – the amount used in our *ex vivo* assays – showed no puromycin labelling when we fed for 1, 6 or 24 h, even if we also fed the larvae bortezomib to prevent any potential proteasomal degradation of labelled peptides *in vivo* (data not shown). We therefore tried a concentration of puromycin which was five times higher. In this case, we did see puromycin labelling after both 6 h and 24 h of feeding, with the longer feeding showing higher levels of labelling (Fig. 1E). However, this labelling was considerably weaker than that seen with the *ex vivo* method, regardless of whether the *ex vivo* labelling was carried out in PBS or media (Fig. 1E). Hence, although feeding of puromycin provides a method for *in vivo* assessment of protein synthesis, it does produce much lower levels of labelling than an *ex vivo* method. Importantly, when we fed the larvae puromycin, we included a blue dye in the food/puromycin mixture. By doing this we could see that the larvae ate the food/puromycin mixture (as observed by blue dye in their guts) and that the amount of feeding was not different compared to when they ate just food alone.

### Effects of nutrient-dependent TORC1 signalling and dMyc on protein synthesis

We next examined whether the puromycin-labelling assay was sensitive to detect changes in proteins synthesis mediated by modulation of known regulators of mRNA translation. In developing larvae, the nutrient-dependent TORC1 kinase pathway is a major regulator of protein synthesis and growth (Grewal, 2009). In nutrient-rich conditions, the TORC1 kinase pathway is activated, and promotes mRNA translation and growth. However, upon nutrient deprivation, the TORC1 pathway is rapidly inhibited and protein synthesis is reduced.

**Fig. 2.**
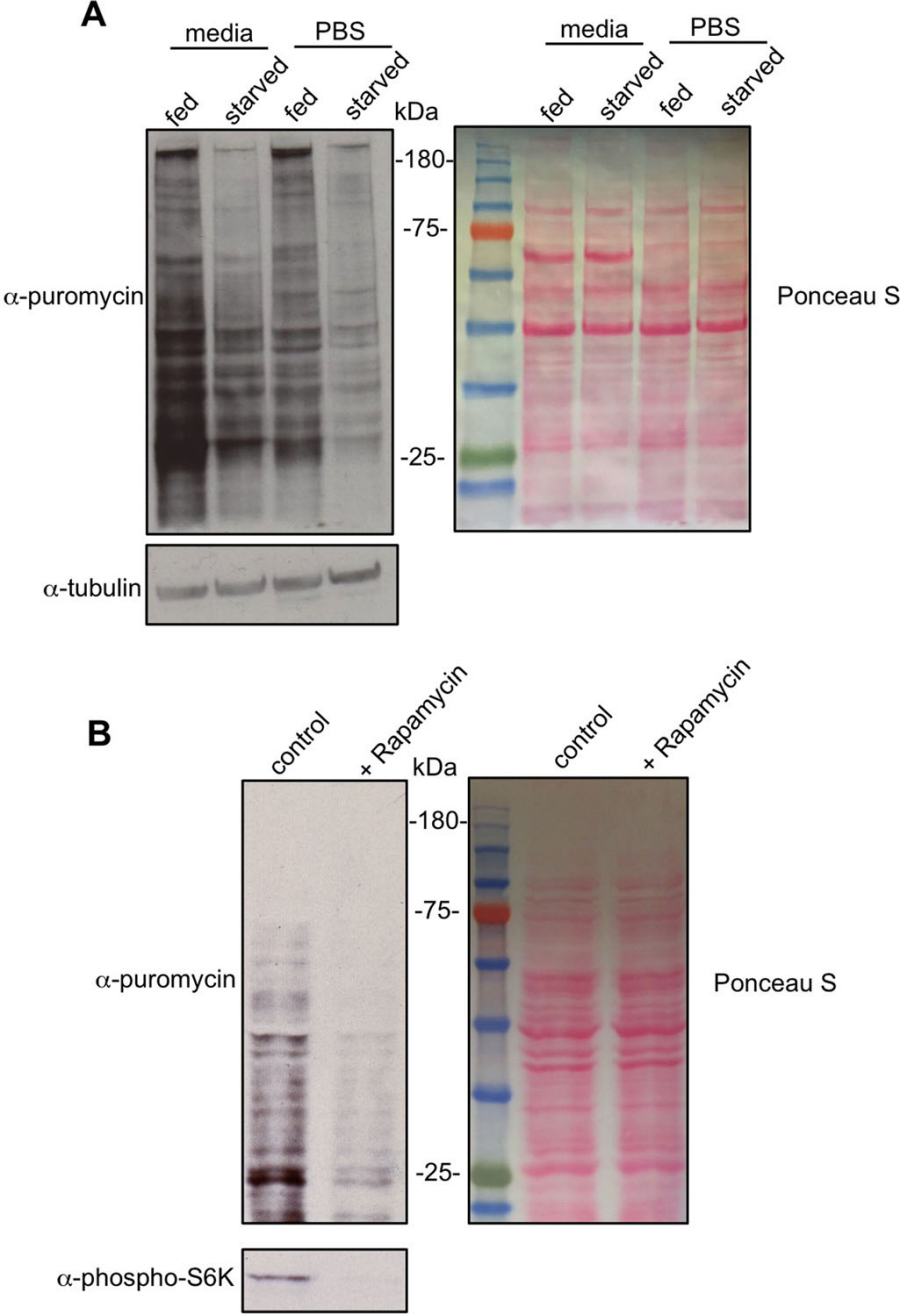
**Regulation of larval protein synthesis by nutrients and TOR signalling.** (A) Fed or 6-h starved third instar larvae were inverted and incubated in either PBS+puromycin (5 µg/ml) or Schneider's media+puromycin (5 µg/ml) for 40 min. Equal amounts of whole larval protein extracts were then analyzed by western blotting. Left, western blot with either anti-puromycin, or anti-tubulin antibodies. Right, Ponceau S staining showing total protein levels. (B) Larvae were inverted and incubated in Schneider's media+puromycin (5 µg/ml) either with DMSO (control) or Rapamycin (20 nM), for 40 min. Equal amounts of whole larval protein extracts were then analyzed by western blotting. Left, western blot with either anti-puromycin, or anti-phospho-S6K antibodies. Right, Ponceau S staining showing total protein levels. All experiments were carried out using *w^1118^* larvae.

We first examined the effects of six-hour nutrient deprivation on protein synthesis in third instar compared to fed controls. We carried out the puromycin labelling in Schneider's media as above and we observed that the starved larvae showed a marked decrease in protein synthesis (Fig. 2A). We reasoned that incubating the starved larvae in Schneider's media (which contains amino acids and glucose) may potentially acutely reverse some of the physiological effects of dietary starvation. Hence we also performed the puromycin labelling by incubating fed versus starved larval tissues in PBS plus puromycin. We saw that the overall level of protein synthesis was lower then when the assay was carried out in Schneider's media. However, as before, we found that starvation led to a marked decrease in protein synthesis (Fig. 2A).

We also looked at pharmacological inhibition of the TORC1 pathway. We carried out the puromycin labelling in third instar larvae and compared the effects of addition or absence of rapamycin – a TOR inhibitor – in the puromycin/media labelling solution. We saw that protein synthesis was markedly reduced when larval tissues were treated with rapamycin (Fig. 2B).

**Fig. 3.**
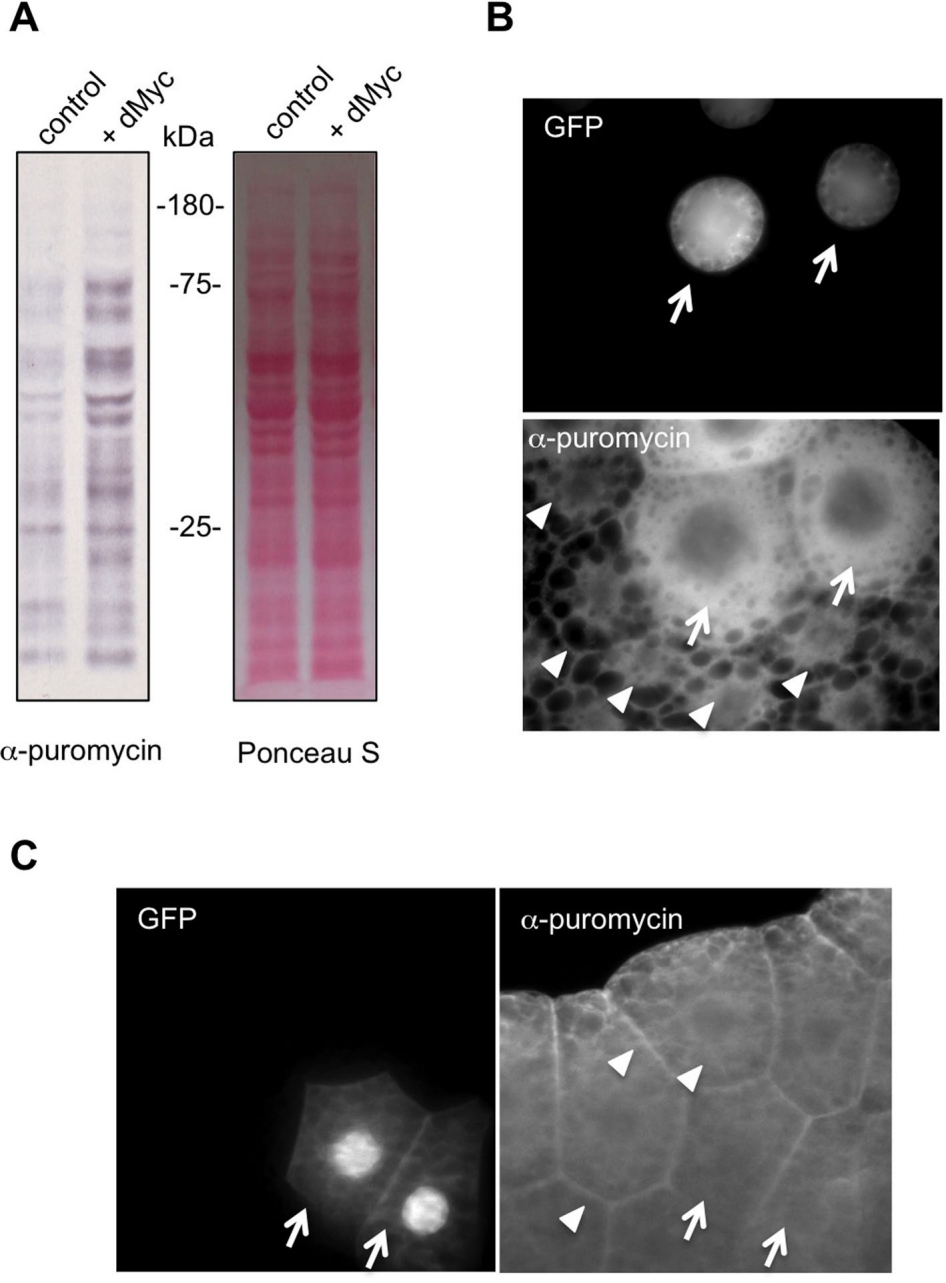
**Regulation of larval protein synthesis by dMyc.** (A) The hsflp-out system was used to induce ubiquitous UAS-dMyc expression in third instar larvae. Control larvae expressed UAS-GFP alone. 24 h following transgene induction, larvae were inverted and incubated in Schneider's media+puromycin (5 µg/ml) for 40 min. Equal amounts of whole larval protein extracts were then analyzed by western blotting. Left, western blot with anti-puromycin antibody. Right, Ponceau S staining showing total protein levels. Genotypes: control=*ywhsflp^122^/+; +/+; act>CD2>GAL4, UAS-GFP/+*, dMyc*=ywhsflp^122^/+; UAS-dMyc/+; act>CD2>GAL4, UAS-GFP/+*. (B) UAS-dMyc clones were generated in larval fat body cells using the flp-out system. Larvae were inverted and incubated in Schneider's media+puromycin (5 µg/ml) for 40 min. Tissues were then immunostained with and anti-puromycin antibody. The nuclear GFP-marked cells overexpressing UAS-dMyc (arrows) show increased puromycin incorporation compared to surrounding non-GFP marked wild-type cells (arrowheads). Genotype: *=ywhsflp^122^/+; UAS-dMyc/+; act>CD2>GAL4, UAS-GFP/+.* (C) UAS-GFP clones were generated in larval fat body cells using the flp-out system. Larvae were inverted and incubated in Schneider's media+puromycin (5 µg/ml) for 40 min. Tissues were then immunostained with an anti-puromycin antibody. The GFP-marked cells overexpressing (arrows) show no change in puromycin incorporation compared to surrounding non-GFP marked wild-type cells (arrowheads). Genotype: *ywhsflp^122^/+; +/+; act>CD2>GAL4, UAS-GFP/+*.

Another regulator of protein synthesis in larvae is the transcription factor dMyc. Overexpression of dMyc increases expression of rRNA, tRNA, and ribosome biogenesis and translation factors in *Drosophila* (Grewal et al., 2005; Steiger et al., 2008; Marshall et al., 2012). We used the hsflp-out system to ubiquitously overexpress dMyc in third instar larvae. We found that dMyc induced a strong increase in protein synthesis compared to control animals (Fig. 3A). We also tested whether the puromycin labelling could be adapted for immunostaining to measure protein synthesis in individual cells. We used the hsflp-out system to generate green fluorescent protein (GFP)-marked cell clones in the larval fat body and then carried out the puromycin-labelling assay but detected puromycin incorporation by immunostaining with the anti-puromycin antibody. We found that dMyc-overexpressing fat body cells showed a marked increase in puromycin labelling compared to surrounding wild-type cells (Fig. 3B). Importantly, we found that cells expressing GFP alone did not show any increase in puromycin labelling (Fig. 3C), suggesting that the increases in puromycin labelling did not simply reflect high levels of transgene expression.

Together these data indicate the utility of the puromycin labelling assay to measure protein synthesis in individual cells or tissues in *Drosophila* larvae.

### Regulation of protein synthesis by hypoxia and heat shock

**Fig. 4.**
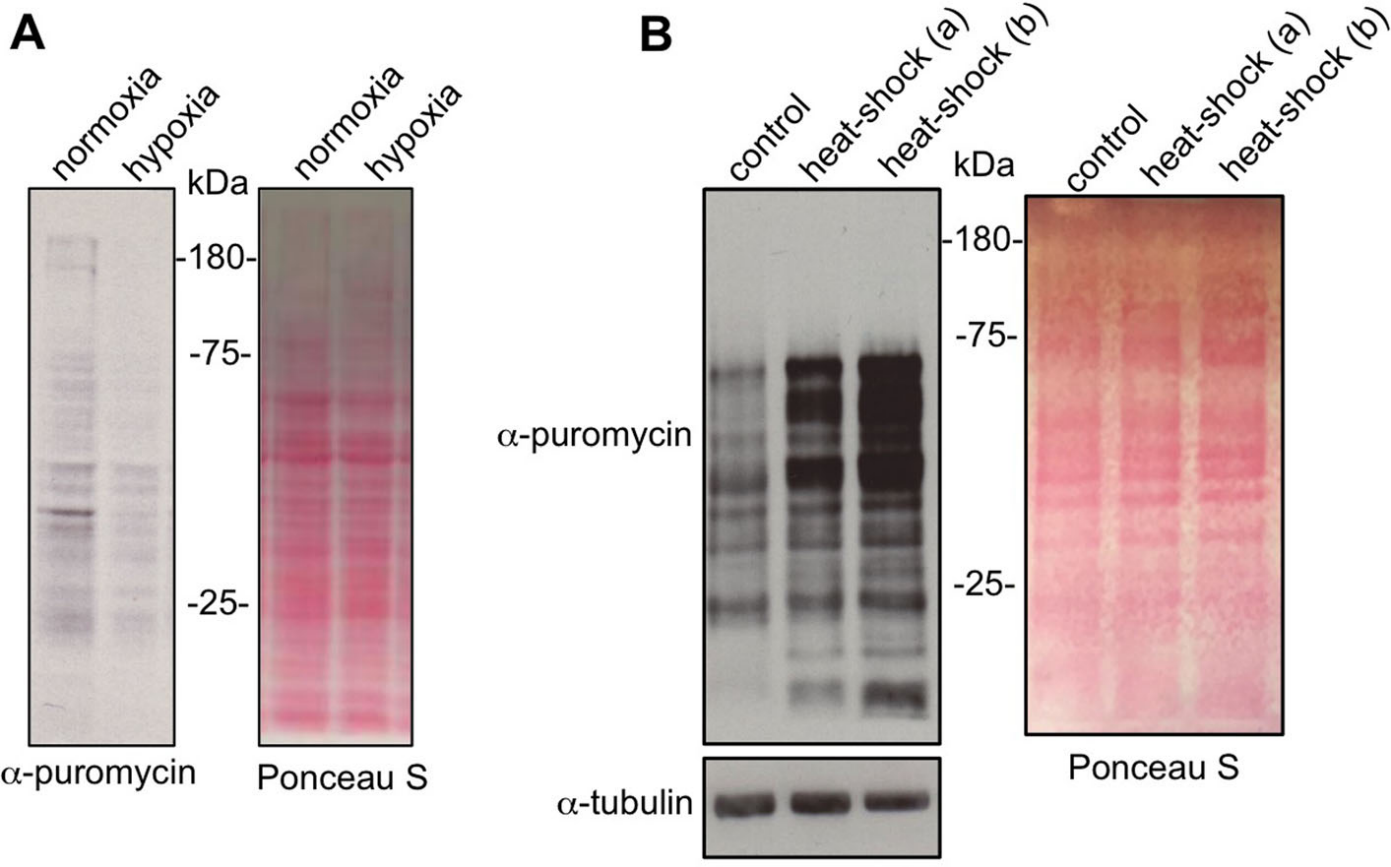
**Regulation of larval protein synthesis by hypoxia and heat stress.** (A) Third instar larvae were either maintained in room air (normoxia) or exposed to 5% O_2_ (hypoxia) for 4 h. Larvae were then inverted and incubated in Schneider's media+puromycin (5 µg/ml) for 40 min. Equal amounts of whole larval protein extracts were then analyzed by western blotting. Left, western blot with anti-puromycin antibody. Right, Ponceau S staining showing total protein levels. (B) Third instar were either maintained at 25°C (control) or exposed to a 1-h 37°C heat shock. Larvae were then inverted and incubated in Schneider's media+puromycin (5 µg/ml) for 40 min. For the heat-shock samples the puromycin incubation was carried out either at room temperature (a) or at 37°C (b). Equal amounts of whole larval protein extracts were then analyzed by western blotting. Left, western blot with anti-puromycin antibody or anti-tubulin antibody. Right, Ponceau S staining showing total protein levels. All experiments were carried out using *w^1118^* larvae.

Exposure of larvae to environmental stress has been shown to affect many conserved signalling pathways known to regulate mRNA translation in other organisms. We therefore used the puromycin labelling assay to examine how two stressors, hypoxia and heat shock, affect protein synthesis in larvae. We first exposed third instar larvae to a 5%O_2_/95%N_2_ mixture for 4 h to induce hypoxia. When we performed puromycin labelling, we saw that the hypoxia-treated larvae showed a marked decrease in protein synthesis (Fig. 4A). We next examined the effect of heat shock on protein synthesis. Third instar larvae were incubated for 1 h at 37°C and then the puromycin labelling was carried out to measure their levels of protein synthesis compared to larvae maintained at 25°C. For the heat-shock conditions, we carried out the 40 min puromycin labelling at both 25°C and 37°C. In both cases, we saw that a 1-h heat shock led to a marked increase in puromycin incorporation in a large number of peptides (Fig. 4B). It is likely that many of these are members of the family of heat-shock proteins that are known to be induced by heat stress.

### Conclusion

We describe a simple and relatively low cost *ex vivo* assay for robust measurement of protein synthesis in larval cells and tissues. The assay can detect both increases and decreases in protein synthesis induced by both genetic and environmental cues. Hence, the assay provides a good alternative to classic approaches to measure protein synthesis such as polysome profiling and ^35^S-methionine labelling. In addition, although Click-IT chemistry has been recently developed with modified analogs of both puromycin and methionine to measure protein synthesis in cells (Liu et al., 2012), these methods are relatively expensive compared to the puromycin labelling approach we describe here. We suggest that the ease of the assay and the ability detect translation in small amounts of tissue will make it a useful approach to monitor how protein synthesis can be regulated in a variety of different growth, developmental and physiological conditions.

## MATERIALS AND METHODS

### Fly stocks

Flies were raised on food with the following composition: 150 g agar, 1600 g cornmeal, 770 g torula yeast, 675 g sucrose, 2340 g D-glucose, 240 ml acid mixture (propionic acid/phosphoric acid per 34 liters water. For all experiments larvae were maintained at 25°C, unless otherwise indicated. The following fly stocks were used: *w^1118^* (used as our ‘wild-type’ stock), *ywhsflp^122^; UAS-dMyc* (Grewal et al., 2005), *ywhsflp^122^; +; +, w; +; act>CD2>GAL4, UAS-GFP*.

dMyc overexpression was achieved using the hsflp-out system. Early third instar larvae were heat shocked at 37°C for 2 h and then returned to 25°C. This is a strong heat-shock that induces flp-mediated recombination in virtually all cells in the larvae, and hence induces ubiquitous expression of dMyc. Puromycin assays were then carried out 24 h later.

### Environmental manipulations

For nutrient starvation, third instar larvae were transferred from fly food to wet filter paper and then left for 6 h. For hypoxia treatments, third instar larvae were transferred to an airtight chamber perfused with a constant flow of 5% oxygen/95% nitrogen for 4 h. During this period, the larvae remained in the food and were eating as normal. For heat-shock experiments, third instar larvae were transferred from 25°C to a 37°C room for 1 h.

### Puromycin assay

Batches of 5-10 larvae were inverted in Schneider's media and then transferred to Eppendorf tubes containing media plus puromycin (Sigma). The larval samples were then left to incubate in a nutator for 40 min at room temperature. For the experiments in Fig. 1A, puromycin was used at the indicated concentrations. For all remaining experiments, puromycin was used at 5 µg/ml. For drug treatments, cycloheximide (100 µg/ml), bortezomib (200 nM) or rapamycin (20 nM, Calbiochem, San Diego, USA) were added to the media/puromycin incubation solution. Following incubation, the inverted larvae were snap frozen (for subsequent western blot analyses) or fixed in paraformaldehyde (for immunostaining). For experiments on specific larval tissues, at the end of the puromycin incubation period, inverted larvae were placed in ice-cold PBS and the relevant tissues were isolated and lysed for western blot analyses.

For the puromycin feeding experiments in Fig. 1E, third instar larvae were transferred to normal food supplemented with 25 µg/ml of puromycin. Larvae were then left to feed for the indicated times (6 or 24 h) before being snap frozen for subsequent western blot analysis.

### Western blotting

Larval tissues were lysed with a buffer containing 20 mM Tris-HCl (pH 8.0), 137 mM NaCl, 1 mM EDTA, 25% glycerol, 1% NP-40 and with following inhibitors: 50 mM NaF, 1 mM PMSF, 1 mM DTT, 5 mM sodium ortho vanadate (Na_3_VO_4_) and protease inhibitor cocktail (Roche cat. no. 04693124001) and phosphatase inhibitor (Roche cat. no. 04906845001), according to the manufacturer's instruction. Protein concentrations were measured using the Bio-Rad Dc Protein Assay kit II (5000112). For each experiment, equal amounts of protein lysates for each sample (usually 15 µg to 40 µg) were resolved by SDS-PAGE and electrotransferred to a nitrocellulose membrane. Blots were then briefly stained with Ponceau S to visualize total protein and then subjected to western blot analysis with specific antibodies. Protein bands were then visualized by chemiluminescence (enhanced ECL solution, Perkin Elmer). Primary antibodies used were anti-puromycin (3RH11) antibody (Kerafast, Boston, USA, cat. no. EQ0001 used at 1:1000), anti-alpha-tubulin (alpha-tubulin E7, *Drosophila* Studies Hybridoma Bank), and anti-phospho-S6K (antibody #9205, Cell Signaling Technology).

#### Immunostaining

Following puromycin incubation, *Drosophila* larvae were fixed in 8% paraformaldehyde/PBS at room temperature for 45 min. After blocking for 2 h in 1% BSA in PBS/0.1% Triton-X 100, inverted larvae were incubated overnight in anti-puromycin antibody (1:1000). Primary antibody staining was detected using Alexa Fluor 488 (Molecular Probes) goat-anti rabbit secondary antibodies. Tissues were then dissected out and mounted on coverslips using mounting media (Vectashield).
